# Acceptance of coronavirus disease 2019 vaccination by cancer patients in Cyprus: A cross-sectional study

**DOI:** 10.1177/10781552211039489

**Published:** 2021-09-25

**Authors:** Zoe Roupa, Maria Noula, Monica Nikitara, Savoula Ghobrial, Evangelos Latzourakis, Giannis Polychronis

**Affiliations:** 1 Department of Life and Health Sciences, 121343 University of Nicosia, Cyprus; 2 112431 Cyprus Anti-cancer Society, Cyprus

**Keywords:** Cancer, SARS-CoV-2, coronavirus disease 2019, vaccine hesitancy, vaccination acceptance

## Abstract

**Introduction:**

The acceptance of an individual to be vaccinated following the introduction of a new vaccine is dependent on multiple factors. Governing factors directing one’s decision to be vaccinated against severe acute respiratory syndrome coronavirus 2, however, are currently unknown and the present study aims at researching these factors within the population of cancer patients.

**Methods:**

A cross-sectional self-administered survey was conducted anonymously between 22 January and 12 February 2021, during the second vaccination phase against severe acute respiratory syndrome coronavirus 2 in Cyprus. The data were collected via an online questionnaire which was formerly used by previously conducted studies. The Mann–Whitney *U* test was applied for the comparison of means between bivariate variables, while the Kruskal–Wallis test was used for the comparison of means in variables with more than two groups. In addition, Spearman correlation coefficients were applied to explore the correlation of continuous variables.

**Results:**

The sample size consisted of a total of 211 cancer patients, 64.9% of which were women with a mean age of 52.6 ± 12.4 years. The findings of the current research indicate a moderate vaccination acceptance among cancer patients (*Μ* = 3.3 ± 0.7, *R* = 1–5).

**Conclusion:**

During the promotion of a vaccine against severe acute respiratory syndrome coronavirus 2 to cancer patients, particular emphasis on specific demographic characteristics, vaccination history and preferred sources of informing the individual are required. In addition, through reviewing the prospective effectiveness and possible outcomes of the specific vaccine according to cancer type and anti-cancer therapy, many of the existing concerns and reservations from cancer patients are expected to be diminished.

## Introduction

Ever since the coronavirus disease 2019 (COVID-19) disease has been declared a pandemic, the severe acute respiratory syndrome coronavirus 2 (SARS-CoV-2) vaccine has been characterised as the most fundamental chance of humanity for the prevention of the spreading of the virus and consequently, the return to normality.^1^ Several international organisations have implemented numerous actions and strategies in their attempt to help accelerate the execution of vaccination campaigns.^2,3^ Subsequently, all European Union countries have developed support tactics for the vaccination campaigns among which is the vaccination priority list for population groups.^3^ Specifically, in Cyprus, on 27 December 2020, the initial vaccination phase began among frontline healthcare workers who come in direct contact with COVID-19 patients as well as residents and employed staff in a care home for older adults. The current schedule for the second vaccination phase aims at the vaccination of individuals 80 years of age and over while the vaccination of cancer patients is planned to take place during the fourth phase of the vaccination schedule.^4^

Given the fact that malignancy and anti-cancer treatments often weaken the immune system, the cancer patient falls within the high-risk group for developing severe illness from the SARS-CoV-2 virus. Consequently, a SARS-CoV-2 infection constitutes a severe threat to cancer patients, their families and their carers alike.^2,3,5,6^

According to Dubé et al.,^7^ the acceptance of an individual to be vaccinated at the introduction of a new vaccine is dependent on several factors (vaccine safety, vaccine efficacy, etc.) which vary according to the civilisation, area and one’s socio-demographic characteristics. What proportion of cancer patients, intent to be vaccinated and which key factors direct their decision for and against their vaccine acceptance (VA)? The current bibliography fails to answer these key questions, leaving a lot of gaps and research limitations following the inevitable pandemic. Taking into consideration the fact that cancer patients’ views are of great significance in their VA, through research, current strategic models of improvement and enrichment of to-date records may become possible.

## Methods and instrument

### Study design and setting

The present study uses a cross-sectional design and is in accordance with the STROBE guidelines.^8^ The research was conducted in Cyprus during the second vaccination phase against the SARS-CoV-2 virus of individuals aged 80 years of age and over. The questionnaire was uploaded for a period of 3 weeks (22 January until 12 February) via an online research tool (questionstar) on the official website of one of the largest non-profit organisations for cancer patients in Cyprus.^9^

### Sampling and sample size

The sample of the present study involved adult individuals who are active or inactive cancer patients and are speakers of the Greek language. In order for the SARS-CoV-2 VA to be recorded in the population under study (>200,000) and for the statistical generalisation of the results to be rendered possible, the minimum estimated sample size was set at 218 individuals. This value has been derived via the use of a sample size calculator^10^ with a margin error of *α* = 5%, confidence interval of 95% and a response distribution of 30%.

### Measurement instrument

Through the existing bibliography, an expected relevant limitation in the measurement tools is detected where researchers with mainly self-improvised measurement scales aim to estimate and interpret the VA regarding the vaccine against SARS-CoV-2. The questionnaire of the present study emerged following the coordination of six experienced researchers and is based on other similar questionnaires within the relevant bibliography.^11–14^ The final survey consists of 22 closed-format questions divided into four parts: (a) four questions concerning participants’ demographic characteristics (age, sex, etc.), (b) five questions relating to vaccination history and intent to be vaccinated (Do you plan to be vaccinated against COVID-19, have you received the influenza vaccine, etc.), (c) five questions using a 4-point Likert scale regarding sources of information and (d) eight questions on VA using a 5-point Likert scale (three questions on vaccine safety and five questions on vaccine importance perceptions).

### Questionnaire validity test

The pilot application was required for the assessment of face validity and internal consistency of the survey in 20 randomly selected cancer patients. No discrepancies in understanding were observed after analysis of the pilot application while the questionnaire showed exceptional internal consistency with a Cronbach’s *α* coefficient of 0.95.^15^

### Statistical analysis

All reported *p* values are two-tailed. Statistical significance was set at *p* < 0.05 and analysis was conducted using the statistical application SPSS (version 26.0). The probability distributions of the reported data were tested via Kolmogorov–Smirnov, while measurement internal consistency was evaluated with the Cronbach’s *α* coefficient. In addition, descriptive data are presented as mean values with standard deviation for continuous variables and as frequencies and percentages for categorical variables.

The Mann–Whitney *U* test was applied for comparison of mean values within continuous and bivariate variables, while the Kruskal–Wallis test was used for the comparison of means of continuous variables and variables with more than two groups. Type I error was controlled via use of the Bonferroni correction, while Spearman correlation coefficients were used to explore the correlation of continuous variables.

Correlation coefficients between 0.1 and 0.3 were set to low, those between 0.31 and 0.5 were set to moderate and those over 0.51 were considered to be high.^16^ Vaccine Acceptance, Vaccine Safety Perception and Vaccine Importance Perception Scales were produced via log transformations. Particularly, scale values between 1.00 and 1.49 were considered not acceptable, those between 1.50 and 2.49 fairly acceptable, any value between 2.50 and 3.49 moderately acceptable while values between 3.50 and 4.49 were acceptable and any value over 4.50 was considered highly acceptable.^17^ Surveys in which the VA questions were not fully completed were considered invalid.

## Results

### Characteristics of the respondents

Respondents’ demographic characteristic, vaccination history and vaccination against SARS-CoV-2 hesitancy are illustrated in Table 1. The sample consists of 211 cancer patients, the majority of which were women (64.9%) with a mean age of 52.6 ± 12.4 years. 10.4% of respondents were cancer patients, employed in a health-related occupational sector. The majority of respondents (49.8%) had a secondary educational background, while only 16.6% of participants underwent an active anti-cancer treatment within the last 2 months. Apparently, 85.3% of respondents were not vaccinated against Influenza A (H1N1) in 2009 and 39.8% of respondents were vaccinated against the seasonal flu within the last 5 years. In addition, 37% of cancer patients reported that they intend to be vaccinated against SARS-CoV-2, 29% reported being unsure while 32.2% reported definitely refuse vaccination. The main reasons for vaccine hesitancy were doubts on vaccine efficacy (36%) in addition to fear of vaccine side effects and the vaccine’s impact on health (36.5%) for cancer patients.

**Table 1.** Demographic data, vaccine history and hesitancy to receive COVID-19 vaccination (*n* = 211).

Variable	*N* (%)	Variable	*N* (%)
Gender		H1N1 vaccinated in 2009	
Male	73 (34.6)	Yes	17 (8.1)
Female	137 (64.9)	No	180 (85.3)
Age	52.6 ± 12.4	Not sure/Don't remember	14 (6.6)
25–39	35 (16.6)	SFV in the last 5 years	
40–54	90 (42.7)	Yes	84 (39.8)
55–69	62 (29.4)	No	126 (59.7)
70–84	24 (11.4)	Not sure/Don't remember	1 (0.5)
Health-related occupations		Want to get COVID-19 vaccinated	
Yes	22 (10.4)	Yes	78 (37.0)
No	188 (89.1)	No	68 (32.2)
Educational background		Not sure	63 (29.9)
Secondary education	105 (49.8)	Hesitancy reasons (MCQ)	
Diploma/Degree	69 (32.7)	Not allowed due to medical history	3 (1.4)
Postgraduate education	37 (17.5)	I have doubts about vaccine efficacy	76 (36.0)
Active anti-cancer treatment		I do not trust the health system	3 (1.4)
Yes	35 (16.6)	I am afraid of the side effects and the impact of the vaccine on my health	77 (36.5)
No	176 (83.4)	Other/Most common answers: 1. I will wait for the oncologist to tell me what he/she thinks. 2. I will let the time pass for now and maybe I will trust it later.	17 (8.1)

H1N1: influenza A virus subtype; MCQ: multiple choice question; SFV: seasonal flu vaccinated.

### Sources of information towards COVID-19 vaccinations

Table 2 demonstrates the percentages and frequencies regarding the sources of information used by respondents towards COVID-19 vaccinations. Particularly, the most frequently used sources were social networks (41.2%) followed by a family member, work colleague or friend (37.9%). Less frequently used sources of information were news broadcasts and mass media (17.5%), healthcare professionals (12.8%) and lastly, official governmental websites (8.1%).

**Table 2.** Sources of information towards COVID-19 vaccinations.

Variable	More use	Often	Sometimes	Less use
	*N* (%)	*N* (%)	*N* (%)	*N* (%)
Healthcare professionals (doctors, nurses, pharmacists, etc.)	27 (12.8%)	31 (14.7)	72 (34.1)	48 (22.7)
News, media (Television, Radio, Newspapers, etc.)	37 (17.5)	44 (20.9)	58 (27.5)	58 (27.5)
Social networks (Facebook, Twitter, YouTube, Instagram, etc.)	87 (41.2)	22 (10.4)	39 (18.5)	45 (21.3)
Official websites (World Health Organization, Portal of the Republic of Cyprus, etc.)	17 (8.1)	31 (14.7)	57 (27)	91 (43.1)
Family member, colleague or friend	80 (37.9)	20 (9.5)	47 (22.3)	43 (20.4)

### Vaccine acceptance

Responses concerning VA for the vaccine against SARS-CoV-2 are recorded in Table 3. More than half of the participants expressed a neutral stance on whether the vaccine contains dangerous ingredients as well as on vaccine safety. Furthermore, only 14.7% of respondents strongly disagreed with the statement that the vaccine side effects are worse than the virus itself. Also, a relatively small percentage (16.1%) believes that the vaccine is important in ending the pandemic while 18% of cancer patients consider that the vaccine is not effective in preventing the COVID-19 disease.

**Table 3.** Vaccine acceptance scale.

Total score Mean ± SD (range)		Variables	Strongly disagree	Disagree	Neutral	Agree	Strongly agree
			*N* (%)	*N* (%)	*N* (%)	*N* (%)	*N* (%)
Vaccine acceptance 3.3 ± 0.7 (1–5)	Vaccine safety perception 3.2 ± 0.8 (1–5)	The vaccine contains dangerous ingredients	26 (12.3)	43 (20.4)	121 (57.3)	10 (4.7)	11 (5.2)
		The vaccine is safe	9 (4.3)	29 (13.7)	114 (54)	50 (23.7)	9 (4.3)
		The side effects of the vaccine are worse than the virus itself	31 (14.7)	42 (19.9)	104 (49.3)	25 (11.8)	9 (4.3)
	Vaccine importance perception 3.4 ± 0.5 (1–5)	The vaccine is important in ending the pandemic	0 (0.0)	13 (6.2)	100 (47.4)	64 (30.3)	34 (16.1)
		The vaccine is effective in preventing the disease	0 (0.0)	38 (18)	93 (44.1)	57 (27)	23 (10.9)
		The vaccine is unnecessary as it targets a harmless disease	45 (21.3)	60 (28.4)	82 (38.9)	23 (10.9)	1 (0.5)
		People to be vaccinated against COVID-19 will play a key role in ending the pandemic	9 (4.3)	9 (4.3)	80 (37.9)	85 (40.3)	28 (13.3)
		To protect public health, we should follow the Government vaccine guidelines	9 (4.3)	41 (19.4)	54 (25.6)	75 (35.5)	32 (15.2)

SD: standard deviation.

In addition, one in five cancer patients disagree strongly with the notion that the vaccine is unnecessary, while the majority (40.3%) considers that individuals who will receive the COVID-19 vaccine will play a fundamental role in ending the pandemic. Lastly, only a small proportion of the respondents (15.2%) are strongly willing to follow the government guidelines for vaccinations and the protection of public health.

In order to yield a total score of VA, the results from the eight relevant questions were added and the result was divided with the Likert scale total statements (five points). Consequently, the score can take a value between 1 and 5 units with the highest score values revealing higher levels of VA. In the same way two other subcategories occurred, vaccine safety perception (range 1–5) and vaccine importance perception (range 1–5) for correlation purposes and subsequently, a greater analytical recording of the issue (Table 3).

The mean score value for VA was 3.3 ± 0.7, while the lowest score occurred at 1.88 units and the highest at 5 units. Regarding the other two subscales, the total score of responses on vaccine safety perception fell within 1–5 units with the mean value at 3.2 ± 0.8 and on vaccine importance perception between 2.2 and 5 units with the mean value at 3.4 ± 0.5 units.

Table 4 illustrates respondents’ VA scores depending on demographic data and vaccine history. Inferential statistics were also applied with the VA score as the dependent variable and the demographic data and vaccine history of participants as the independent variables.

**Tablet 4.** Distribution of VA score depending on demographic data and vaccine history.

Variable	Mean ± SD	Mann–Whitney *U* test	SE^a^	*p*
Gender		3204.50		0.00
Male	3.6 ± 0.6		0.076	
Female	3.2 ± 0.7		0.066	
Age		10.46^b^		0.01
25–39	3.4 ± 0.6		0.111	
40–54	3.2 ± 0.6		0.068	
55–69	3.5 ± 0.9		0.122	
70–84	3.5 ± 0.6		0.130	
Health-related occupations		1299.50		0.04
Yes	3.8 ± 0.6		0.147	
No	3.3 ± 0.7		0.055	
Educational background		41.16^b^		0.00
Secondary education	3 ± 0.6		0.064	
Diploma/degree	3.5 ± 0.7		0.092	
Postgraduate education	3.9 ± 0.5		0.092	
Active anti-cancer treatment		2587.5		0.13
Yes	3.4 ± 0.6		0.103	
No	3.3 ± 0.7		0.059	
H1N1 vaccinated in 2009		29.69^b^		0.00
Yes	4.1 ± 0.6		0.154	
No	3.2 ± 0.7		0.054	
Not sure/don't remember	4 ± 0.4		0.126	
SFV in the last 5 years		2302.00		0.00
Yes	3.8 ± 0.6		0.075	
No	3 ± 0.6		0.059	
Not sure/don't remember	–		–	
Want to get COVID-19 vaccinated		134.57		0.00
Yes	4.1 ± 0.4		0.054	
No	2.7 ± 0.4		0.057	
Not sure	3.1 ± 0.4		0.056	

H1N1: influenza A virus subtype; SD: standard deviation; SE: standard error; SFV: seasonal flu vaccinated.

^a^ Standard error of the estimate.

^b^ Kruskal–Wallis test.

The VA score was found to deviate significantly according to the demographic characteristics of the sex, age and educational background and to whether the respondents’ occupation was related to the healthcare sector. In particular, men respondents were more willing to accept the vaccine as their VA score was 0.4 units higher than women respondents. Regarding the age variable, following the Bonferroni correction for multiple tests, participants falling within the 40–54 and 55–69 age groups had a lower VA score in relation to any other age group (*p* = 0.02). Likewise, after applying the Bonferroni correction, the educational background was found to differ considerably in all three categories since those with a postgraduate education had a higher acceptance score by 0.4 units compared with those with a diploma/degree and by 0.9 units to those with a secondary education. Moreover, when the occupation of the cancer patient was related to the healthcare sector, a higher vaccination against COVID-19 acceptance score resulted by 0.5 units.

According to our data, the VA score is not affected by whether the patient is in active anti-cancer treatment (*p* = 0.13). Statistical significance differences are apparent however, according to vaccination history. Specifically, applying the Bonferroni correction, if the participant was not vaccinated in 2009 against the influenza A (H1N1) virus results in a reduced COVID-19 VA in comparison with both participants who were vaccinated in 2009 (*p* = 0.00) and those who were unsure/did not remember whether they were vaccinated in 2009 (*p* = 0.00). Statistical significance difference (*p* = 0.00) is also revealed in individuals who received the vaccine against the seasonal flu within the last 5 years (*M* = 3.8 ± 0.6) when compared with those who were not vaccinated (*M* = 3 ± 0.6).

In Table 5, the vaccine safety perception scores are given together with the vaccine importance perception scores according to the independent variable regarding respondents’ willingness to be vaccinated against COVID-19.

**Table 5.** Distribution of willingness to be vaccinated against COVID-19 with VA, safety perception and importance perception scores.

Variable	Mean ± SD	Mann–Whitney *U* test	SE^a^	*p*
Want to get COVID-19 vaccinated				
		106.05^b^ 137.48^c^		0.00^b^ 0.00^c^
Yes	3.9 ± 0.6^b^ 4.2 ± 0.4^c^		0.073^b^ 0.052^c^	
No	2.6 ± 0.7^b^ 2.7 ± 0.5^c^		0.085^b^ 0.061^c^	
Not sure	2.9 ± 0.9^b^ 3.3 ± 0.4^c^		0.067^b^ 0.059^c^	

SD: standard deviation; SE: standard error.

^a^ Standard error of the estimate.

^b^ According to the vaccine safety perception score.

^c^ According to the vaccine importance perception score.

According to the vaccine safety scores produced after the Bonferroni correction, there seems to be no statistical significance difference (*p* = 0.33) between respondents who do not support vaccination against SARS-CoV-2 (*M* = 2.7 ± 0.5) and respondents who are unsure of whether to be vaccinated against SARS-CoV-2 (*M* = 2.9 ± 0.9). On the contrary, there is a considerable difference (*p* = 0.00) in cancer patients who are in favour of vaccination (*M* = 3.9 ± 0.6) in comparison with the other two aforementioned categories. Our vaccine importance perception scores show significant differences when compared with all three category groups even after applying the Bonferroni correction. Specifically, respondents who intend to be vaccinated scored 1.5 units higher in vaccine importance perception in relation to vaccine refusers and 0.9 units higher compared with those who were unsure.

Table 6 illustrates the correlation coefficient of age and sources of information with the relevant VA score.

**Table 6.** Correlations with VA score.

		Vaccine acceptance
Age	*r*_s_	0*.*14
	*p*	0*.*04
Sources of information		
Healthcare professionals	*r*_s_	0*.*36
	*p*	0*.*00
News, media	*r*_s_	0*.*50
	*p*	0*.*00
Social networks	*r*_s_	−0*.*59
	*p*	0*.*00
Official websites	*r*_s_	0*.*35
	*p*	0*.*00
Family member, colleague or friend	*r*_s_	−0*.*54
	*p*	0*.*00

The results show a weak positive correlation between VA and the age of participants (*r*_s_ = 0.14, *p* = 0.04). Consequently, as the participants’ age increases so does the possibility of the cancer patient to be in favour of vaccination. Additionally, our results on sources of information and VA scores indicate several correlations. In particular, moderate positive correlations were found since the possibility for VA increases when cancer patients use the official websites as their main information sources (*r*_s_ = 0.35, *p* = 0.00) and/or the mass media (*r*_s_ = 0.50, *p* = 0.00) and/or healthcare professionals (*r*_s_ = 0.36, *p* = 0.00). At the same time, a significant negative correlation is present when the sources of information involve the social networks (*r*_s_ = −0.59, *p* = 0.00) and/or a family member, colleague or friend (*r_s_* = −0.54, *p* = 0.00) as this decreases the possibility for VA.

## Discussion

The findings of the present study indicate a moderate VA the COVID-19 vaccine, while only one in three cancer patients from the total sample (*N* = 211), reported to be in favour of vaccination. Subsequently, cancer patients who support vaccination scored higher in both vaccine safety perception and in vaccine importance perception in relation to both vaccine refusers and to those who reported unsure. Patients who refuse vaccination or are unsure, express several doubts and reservations. Particularly, the main reasons to refuse or to be unsure of vaccination were uncertainties for vaccine efficacy, fear of side effects and vaccine effects on health. In addition, according to Fisher et al.,^18^ inadequate trust in the government and the country’s health system constitutes some main causes against VA. In a similar study conducted in France, by Barrière et al.,^19^ the main reasons reported among patients who were in favour of vaccination were fear about their health, the desire to protect their relatives, the duty for collective responsibility and finally the wish to return to a normal life. Consequently, during the development of strategies and vaccination promotion campaigns among cancer patients, all reported reservations and doubts concerning the vaccine must be taken into consideration.

Both the study conducted by Barrière et al.,^19^ as well as the current study, concluded that VA is, in fact, affected by demographic factors and the vaccination history of cancer patients. On the contrary, results of the current research indicate that VA seems to be unaffected by whether the patient is in active or inactive anti-cancer treatment. However, men, postgraduate education, a health-related occupation and the increase in the age of the individual constitute determining demographic factors in increasing VA. Another above-mentioned determining factor in supporting vaccination among patients is vaccination history. In particular, when the cancer patient had received the vaccine against the Influenza A (H1N1) virus or if he/she had been vaccinated the last 5 years against the seasonal flu a higher VA was reported.

The most common source of information among respondents for vaccination was the social networks (41.2%) while the least common one was the official governmental websites (8.1%). Furthermore, VA was found to lessen as the respondent is mainly informed via the social networks or via close contacts but was found to increase as he/she is using as main sources of information the official websites, mass media and/or healthcare professionals.

## Limitations

Despite the fact that the study’s percentage response (82%) was satisfactory, the distribution of the questionnaire via the internet and the social media, however, increases the possibility for selection bias. Furthermore, allocation bias may have been the case with only two participants of the study (83 and 84 years old) since the questionnaire could have been completed between 22 January and 12 February 2020 which falls within a period where individuals <80 years of age could have been vaccinated.^4^ Moreover, the use of a new measurement scale for the results which has not been previously tested may increase the likelihood of Information Bias.^20^ Consequently, all limitations acknowledged here are important factors that must yield some reservations when applying the generalisation of the results of the present study.

## Conclusions

The present findings demonstrate a moderate VA against SARS-CoV-2 on behalf of cancer patients. No correlation between willingness to be vaccinated and whether the individual is in active anti-cancer treatment was found. However, during the promotion of the COVID-19 vaccine the focus particularly in the female population, in individuals with a degree or secondary education, individuals with a non-healthcare professional background, those who are mainly informed via social networks or close contacts as well as individuals with an inactive vaccine history is recommended.

Future research studies conducted with a larger sample, examining the vaccine efficacy and possible health effects according to cancer type and anti-cancer treatments could possibly limit existing reluctances and concerns from many cancer patients regarding VA.
